# Audit for prescription and administration of PRN buccal midazolam for people with learning disabilities and epilepsy in the Hertfordshire & Essex

**DOI:** 10.1192/bjo.2021.588

**Published:** 2021-06-18

**Authors:** Anu Sharma, Indermeet Sawhney

**Affiliations:** 1 Saffron Ground, Ditchmore lane; 2 Tekhnicon House, Springwood Drive

## Abstract

**Aims:**

The current audit aims to identify the gaps in the practice of administering prn buccal midazolam, for management of epilepsy in people with intellectual disability and to review training needs, with a view to improve patient care.

**Background:**

Convulsive status epilepticus is a medical emergency requiring admission to hospital and has a mortality as high as 20% (SUDEP -Sudden Unexpected Death in Epilepsy). It is imperative that the carers are fully aware of the risks associated with the epileptic attacks, are able to recognize the attacks and offer rescue medication to the patient in a timely and effective manner. National guidelines have been drafted jointly by ESNA (Epilepsy Specialist Nurses Association) and ILAE (International League Against Epilepsy) for prn administration of buccal midazolam. The use of rescue medication by trained carers can significantly improve the outcome and reducing the risk of hospital administration and chances of SUDEP. Buccal midazolam is widely used to manage prolonged seizures. Administration should be undertaken only by people who have received both epilepsy awareness and buccal midazolam training.

**Method:**

All patients with intellectual disability with epilepsy were studied and patients who were prescribed prn midazolam have been shortlisted shortlisted for data analysis. A template was designed and data are being collected from the carers, community nurses and the prescribing clinicians. Data are categorized under headings of background information about epilepsy, recognizing complications during a seizure and the ability to administer buccal midazolam. Further information about the type and the frequency of training courses (including the refresher courses) being offered to the carers for administration of buccal midazolam would also be documented. There is also data collection pertaining to the timely review of the care plan by the prescribing clinicians and the about the licenced or unlicensed used of midazolam. The quality of epilepsy awareness training is being explored in depth and also the competency of the county council nurses offering the training to the carers shall be analysed under different headings.

**Result:**

Data are currently being collected in Hertfordshire and Essex mental health services.

**Conclusion:**

After the data analysis, the short comings shall be addressed and effective measures shall be put in place to improve the quality of training being provided to the carers and to standardise the practice for prn midazolam prescription.

